# Aliskiren Attenuates Steatohepatitis and Increases Turnover of Hepatic Fat in Mice Fed with a Methionine and Choline Deficient Diet

**DOI:** 10.1371/journal.pone.0077817

**Published:** 2013-10-21

**Authors:** Kuei-Chuan Lee, Che-Chang Chan, Ying-Ying Yang, Yun-Cheng Hsieh, Yi-Hsiang Huang, Han-Chieh Lin

**Affiliations:** 1 Division of Gastroenterology, Department of Medicine, Taipei Veterans General Hospital, Taipei, Taiwan; 2 Department of Medicine, National Yang-Ming University School of Medicine, Taipei, Taiwan; 3 Institute of Clinical Medicine, National Yang-Ming University School of Medicine, Taipei, Taiwan; 4 Division of General Medicine, Department of Medicine, Taipei Veterans General Hospital, Taipei, Taiwan; Centro de Investigación en Medicina Aplicada (CIMA), Spain

## Abstract

**Background & Aims:**

Activation of the renin-angiotensin-system is known to play a role in nonalcoholic steatohepatitis. Renin knockout mice manifest decreased hepatic steatosis. Aliskiren is the first direct renin inhibitor to be approved for clinical use. Our study aims to evaluate the possible therapeutic effects and mechanism of the chronic administration of aliskiren in a dietary steatohepatitis murine model.

**Methods:**

Male C57BL/6 mice were fed with a methionine and choline-deficient (MCD) diet to induce steatohepatitis. After 8 weeks of feeding, the injured mice were randomly assigned to receive aliskiren (50 mg_·_kg^-1^ per day) or vehicle administration for 4 weeks. Normal controls were also administered aliskiren (50 mg_·_kg^-1^ per day) or a vehicle for 4 weeks.

**Results:**

In the MCD mice, aliskiren attenuated hepatic steatosis, inflammation and fibrosis. Aliskiren did not change expression of lipogenic genes but increase turnover of hepatic fat by up-regulating peroxisome proliferator-activated receptor α, carnitine palmitoyltransferase 1a, cytochrome P450-4A14 and phosphorylated AMP-activated protein kinase. Furthermore, aliskiren decreased the hepatic expression of angiotensin II and nuclear factor κB. The levels of oxidative stress, hepatocyte apoptosis, activation of Kupffer cells and hepatic stellate cells, and pro-fibrotic markers were also reduced in the livers of the MCD mice receiving aliskiren.

**Conclusions:**

Aliskiren attenuates steatohepatitis and fibrosis in mice fed with a MCD diet. Thus, the noted therapeutic effects might come from not only the reduction of angiotensin II but also the up-regulation of fatty acid oxidation-related genes.

## Introduction

Nonalcoholic fatty liver disease (NAFLD), which is characterized by an increase in intrahepatic triglyceride content with inflammation (non-alcoholic steatohepatitis, NASH) or without inflammation (simple steatosis), is rapidly emerging as the most prevalent hepatic disorder in the Western world [1] with NASH possibly progressing to liver cirrhosis and hepatocellular carcinoma [2]. It has been suggested that the renin-angiotensin system (RAS) plays a role in NAFLD/NASH [3–5]. Further, transgenic hypertensive rats overexpressing the mouse renin gene with elevated tissue angiotensin II (Ang II) developed hepatic steatosis, inflammation and fibrosis [6], whereas the mice lacking the renin gene fed with high fat diet stored decreased fat in the liver [7]. Therefore, in light of these findings, direct renin inhibition provides a logical method of treating NAFLD by completely blocking RAS activity. 

Aliskiren, the first in the class of direct renin inhibitors, has recently been approved for clinical use. It links to the active site within renin [8] that is responsible for the hydrolysis of the Leu10-Val11 bond of angiotensinogen, which leads to the generation of the decapeptide fragment angiotensin I. This blockade of the enzyme activity of renin leads to lowered plasma renin activity and then decreased plasma angiotensin I and II [9]. Not only did we recently find aliskiren attenuated liver injury in chronic carbon tetrachloride injured mice [10], but it has also been observed that aliskiren improved insulin resistance and lipid abnormality in mice [11] and transgenic Ren2 rats [12] [13]. Thus, the aims of this study were to investigate whether aliskiren can provide therapeutic effects in a dietary steatohepatitis murine model and to elucidate the underlying mechanism involved. 

## Materials and Methods

### Animals

Adult male C57BL/6 mice aged 8-10 weeks (BioLasco Taiwan Co., Ltd, Taipei, Taiwan) were used in all experiments. All mice were caged at 22°C, with a 12-hour light-dark cycle, and allowed free access to food and water. All animals received humane care in accordance with *Guide for the care and Use of Laboratory Animals* (published by National Institutes of Health). The experiment had been approved by the animal ethical committee of Taipei Veterans General Hospital (IACUC number: 2011-215, approved on 02/Jan/2012). 

### Protocol

Chronic liver injury was induced by feeding the mice a methionine choline-deficient (MCD) diet (n=14). Mice fed with a methionine-choline-supplemented diet served as normal controls (n=13). After being fed with an 8-week MCD diet, the MCD mice were randomly divided to receive aliskiren (the MCD-Ali group, n=7) or vehicle (the MCD-V group, n=7). The normal control mice were also administered aliskiren (the N-Ali group, n=7) or vehicle (the N-V group, n=6). ALZET osmotic mini pumps (DURECT Corporation, Cupertino, California, U.S.A.) filled with aliskiren (provided from Novartis, 50 mg_·_kg^-1^ per day) or the vehicle (double distilled water 100 μL) were implanted subcutaneously in the MCD or normal mice for 4 weeks. The dose of aliskiren used had been shown to exert sufficient inhibition of the renin activity in the mice of previous studies [14]. 

Four weeks after randomization, all groups of mice were sacrificed after overnight fasting. Plasma samples were collected and processed immediately or stored at -80°C until assay. The liver and spleen were rapidly excised and weighed after ice cold phosphate buffered saline perfusion. Aliquots of liver were snap frozen in liquid nitrogen and kept at -80°C until being analyzed. A portion of the liver was fixed in 10% formalin for histology. 

### Measurements of blood biochemistry, angiotensin II and insulin

The blood biochemistry was measured using a standard auto-SMAC analyzer (Roche Diagnostics GmbH, Mannheim, Germany). The plasma angiotensin II and insulin levels were determined according to the AssayMax Angiotensin II ELISA kit (AssayPro Co., MO, U.S.A.) and Mouse Insulin ELISA Kit (Millipore, MA, U.S.A.) respectively. 

### Measurements of triglyceride content and thiobarbituric acid reactive substances (TBARS) in livers

The triglyceride content in livers was measured by a Triglyceride Quantification Kit (Abcam, MA, U.S.A.); the TBARS in livers were measured by using a TBARS Assay Kit (Cayman Chemical, MI, U.S.A.) according to the manufacturers’ guidance. 

### Real-time quantitative reverse transcriptase–polymerase chain reaction (QPCR)

Complementary DNAs were synthesized by reverse transcription of 1µg total RNA according to the MMLV reverse transcriptase 1st-strand cDNA Synthesis Kit (EPICENTRE, Madison, WI, U.S.A.). The primers used are listed in Table S1 in File S1. Quantitative gene expression was performed on an ABI PRISM 7900HT Sequence detection system (Applied Biosystems Inc., Foster City, CA, U.S.A.) using SYBR Green technology. 

### Western blot analysis

The Western blotting was performed as previously described [10]. The blots were incubated with the antibodies listed in Table S2 in File S1. The blots were developed by enhanced chemiluminescence (Amersham ECL Western Blotting Analysis System). The intensities of the bands of interest were analyzed by a densitometric analysis program (Kodak Digital Science KDS 1D 20).

### Histological examinations

Masson’s trichrome stain and oil red O stain were used to evaluate the degree of collagen deposition and steatosis, respectively. 

The terminal deoxynucleotide transferase mediated dUTP nick-end labeling (TUNEL) assay was performed to evaluate apoptosis by an *in situ* cell death detection kit (Roche Diagnostics GmbH, Mannheim, Germany). 

For immunohistochemistry staining, the tissue sections underwent antigen retrieval (proteinase K for F4/80 and phospho-p47 phox, pH 8.0, 25°C; 10 mmol_·_L^-1^ sodium citrate buffer solution, pH 6.0, 95°C, 30 min for others) and blocking (3% H_2_O_2_, 15 min; 5% heated bovine serum albumin, 60 min) were incubated at 4°C overnight with primary antibodies (Table S2 in File S1). After being washed, the slides were incubated with appropriate horseradish peroxidase polymer-conjugated secondary antibodies (Biocare Medical, Concord, CA, U.S.A.) for 30 min at room temperature. The slides were colored using 3,3'-diaminobenzidine (Dako, Inc., Carpinteria, CA, U.S.A.) and counterstained with Mayer’s haematoxylin for 30 sec. For immunofluorescent staining, the tissues were incubated with goat anti-rabbit DyLight 488 (Biocare Medical, Inc.) for 120 min at 37°C on day 2 and counterstained with 4',6-diamidino-2-phenylindole (Cell Signaling). 

For quantification purposes, five fields per slide were randomly selected. The percentages of positive stained areas were measured using Image-Pro Plus (Media Cybernetics, Inc., Silver Spring, MD, U.S.A.) in an independent manner. The average of the five fields in each slide was then calculated.

### Statistical analysis

Data were analyzed by GraphPad Prism 4 (GraphPad Software, San Diego, CA, U.S.A.) and expressed as means ± standard error of the mean. ANOVA followed by the post hoc Newman-Keuls test or Mann-Whitney U-test when appropriate was used for comparisons between groups. Significance was determined at a p-value <0.05.

## Results

### Aliskiren attenuated steatosis and inflammation in MCD mice

**Figure 1:**
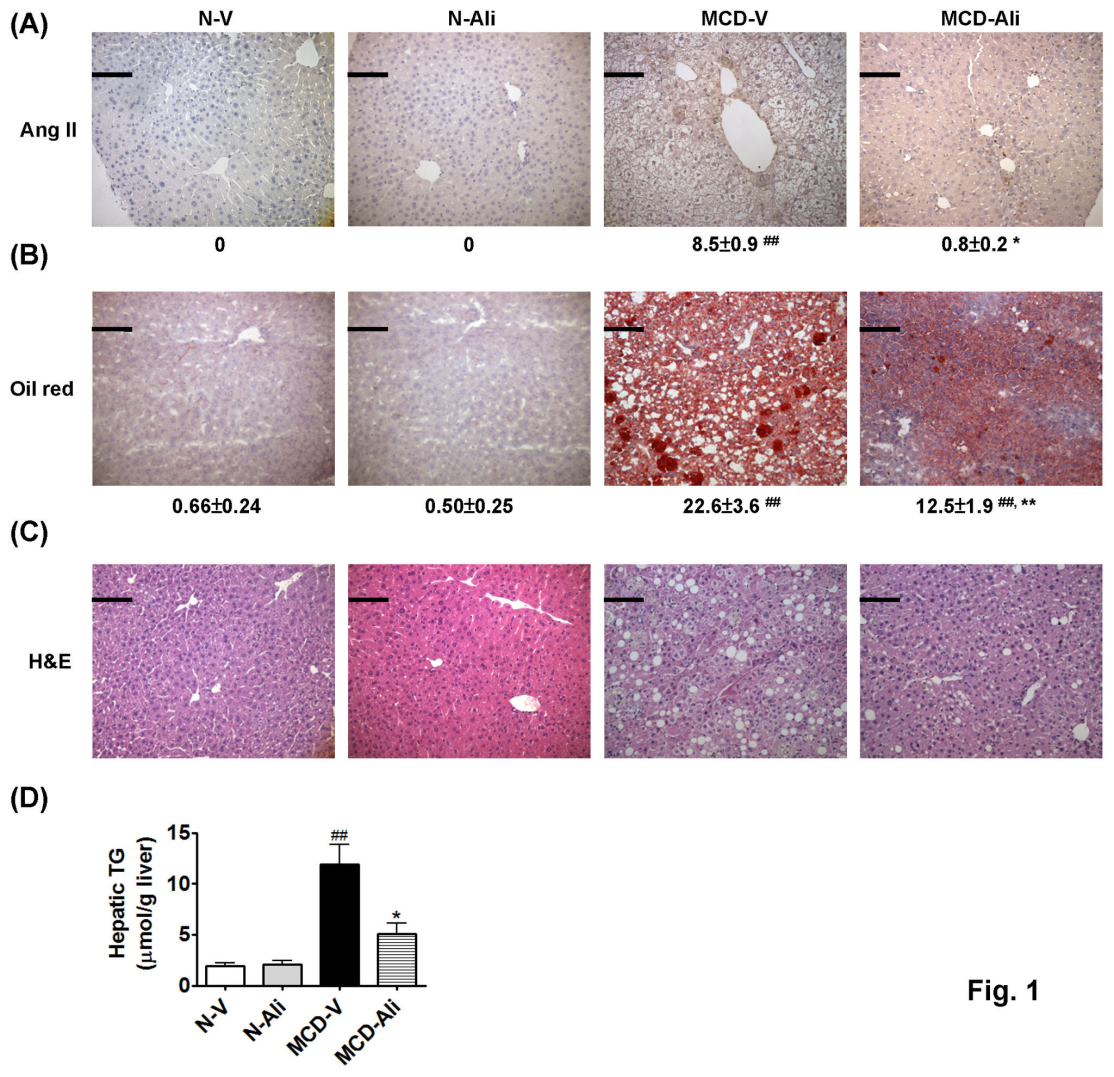
Aliskiren (Ali) decreased expression of angiotensin II (Ang II) and attenuated steatosis and inflammation in the livers of mice fed with methionine choline deficiency (MCD) diet. (**A**) Representative immunohistochemistry photomicrographs (×200) of hepatic Ang II and (**B**) the oil red O stain in all groups with quantification of the positive stained areas to the below. Scale bar = 100 μm. The haematoxylin and eosin (H&E) stain (**C**) and hepatic triglyceride (TG) content (**D**) in four groups. N-V/N-Ali: normal (N) mice receiving vehicle (V) or aliskiren; MCD-V/MCD-Ali: MCD mice treated with vehicle or aliskiren (##: P<0.01 vs. N-V; *: P<0.05 vs. MCD-V; **: P<0.01 vs. MCD-V).

The systemic and hepatic Ang II levels were increased significantly in the MCD-V mice and decreased in the MCD-Ali mice (Table 1, Figure 1A). The plasma levels of alanine aminotransferase and aspartate aminotransferase were significantly elevated in the MCD mice compared with the normal mice (Table 1). After administration of aliskiren, the MCD-Ali mice had significant reduction in plasma levels of alanine aminotransferase (p=0.048) and aspartate aminotransferase (P=0.017) compared with the MCD-V group. 

**Table 1:** Blood biochemistry, and weights of body, liver and spleen in all groups.

Group	N-V (n=6)	N-Ali (n=7)	MCD-V (n=7)	MCD-Ali (n=7)
ALT (U•L^-1^)	67.5±5.1	54.3±9.5	587.0±200.4 ^##^	210.0±40.2 ^##^, *
AST (U•L^-1^)	95.8±11.1	115.7±19.9	728.6±95.1 ^##^	422.1±55.1 ^##^, *
Ang II (pg/ml)	26.25±3.99	19.22±1.48	81.67±4.39 ^##^	58.25±2.01^##,^ **
BW (gm)	28.9±0.35	28.58±0.69	13.5±0.48 ^##^	13.83±0.21^##^
LW (gm)	1.398±0.06	1.47±0.04	0.80 ± 0.05 ^##^	0.763±0.03 ^##^
SW (gm)	0.094±0.007	0.081±0.005	0.03±0.002 ^##^	0.03±0.001 ^##^

All data are expressed as the mean ± SEM. ALT: alanine aminotransferase, AST: aspartate aminotransferase, Ang II: angiotensin II, BW: body weight, LW: liver weight, SW: spleen weight, MCD: methionine choline-deficient, N-V/N-Ali: normal (N) mice receiving vehicle (V) or aliskiren; MCD-V/MCD-Ali: MCD mice treated with vehicle or aliskiren. ## P<0.01 vs. N-V; * P<0.05 vs. MCD-V; ** P<0.01 vs. MCD-V group.

The hepatic triglyceride content was significantly higher in the MCD-V group (11.9 ± 1.9 μmol/g liver) than in the N-V group (1.7 ± 0.3 μmol/g liver, p<0.01). Aliskiren treatment decreased hepatic triglyceride levels significantly in the MCD-Ali mice (5.1 ± 1.1 μmol/g liver, p<0.01 vs. the MCD-V group) (Figure 1D). The oil red O staining of livers showed similar results (Figure 1B). 

In the hematoxylin and eosin stain (Figure 1C), the severity of NAFLD was evaluated by using the NAFLD activity score [15], which shows that the MCD-V group developed significantly increased steatosis, inflammatory foci and hepatocyte ballooning compared with the N-V group. Aliskiren treatment significantly reduced these injuries (Table 2). 

**Table 2:** Components of NAFLD activity score and fibrosis staging.

Group	N-V (n=6)	N-Ali (n-7)	MCD-V (n=7)	MCD-Ali (n=7)
Steatosis	0	0	0.91±0.13 ^##^	0.32±0.11**
Lobular Inflammation	0	0	2.41±0.15 ^##^	1.69±0.18 ^##^,**
Hepatocyte Ballooning	0	0	1.54±0.22 ^##^	0.17±0.11 **
Fibrosis stage	0	0	2.38±0.26 ^##^	1.30±0.18 ^##^,**

All data are expressed as the mean ± SEM. NAFLD: non-alcoholic fatty liver disease, MCD: methionine choline-deficient, N-V/N-Ali: normal (N) mice receiving vehicle (V) or aliskiren, MCD-V/MCD-Ali: MCD mice treated with vehicle or aliskiren. ## P<0.01 vs. N-V; ** P<0.01 vs. MCD-V group.

In in vitro experiment, the HepG2 cells incubated with Ang II (10^-7^M) had more steatosis than the vehicle group (p=0.015) (Figure S1A in File S1). Treatment with both of aliskiren (10^-5^M) and Ang II (10^-7^M) significantly reduced steatosis when compared with the HepG2 cells incubated with Ang II (10^-7^M) alone (p=0.036). 

### Aliskiren increased insulin sensitivity

The plasma levels of glucose and insulin in the MCD-V and MCD-Ali groups were significantly lower than those in the N-V group. The levels of quantitative insulin sensitivity check index (QUICKI) in the MCD mice with or without aliskiren treatment were higher than those in the normal mice (Table 3). Aliskiren treatment further decreased plasma glucose and insulin levels with elevated QUICKI in the MCD-Ali mice (p=0.018, 0.053, 0.018 vs. the MCD-V group respectively).

**Table 3:** Fasting plasma glucose, insulin and QUICKI levels.

Group	N-V (n=6)	N-Ali (n=7)	MCD-V (n=7)	MCD-Ali (n=7)
Glucose (mg/dl)	180.0±6.7	181.4±11.6	80.0±12.1 ^##^	33.6±11.5 ^##^, *
Insulin (ng/ml)	0.53±0.07	0.44±0.03	0.31±0.04 ^##^	0.25±0.01 ^##^
QUICKI	0.52±0.02	0.53±0.01	0.80±0.09 ^##^	1.61±0.29 ^##^, *

All data are expressed as the mean ± SEM. QUICKI: quantitative insulin sensitivity check index, a measurement of insulin sensitivity derived from the formula 1/ (log insulin ╳ glucose), MCD: methionine choline-deficient, N-V/N-Ali: normal (N) mice receiving vehicle (V) or aliskiren, MCD-V/MCD-Ali: MCD mice treated with vehicle or aliskiren. ## P<0.01 vs. N-V; * P<0.05 vs. MCD-V.

### The effect of aliskiren on factors regulating fatty acid metabolism

**Figure 2:**
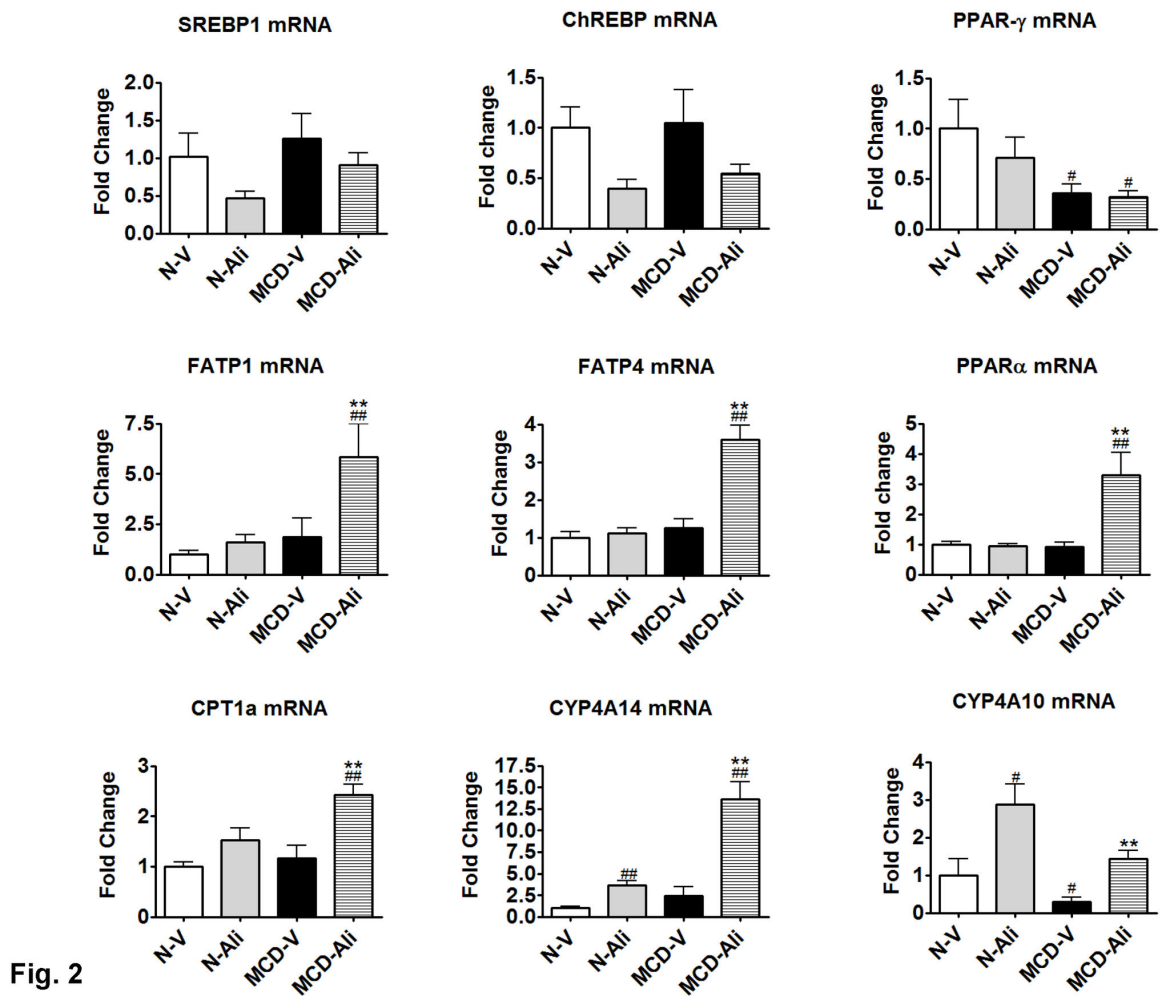
Aliskiren increased turnover of triglyceride. The transcript expression of sterol regulatory element binding protein-1 (SREBP1), carbohydrate responsive element binding protein (ChREBP), peroxisome proliferator-activated receptor gamma (PPAR-γ), fatty acid transport protein 1 and 4 (FATP1, FATP4), peroxisome proliferator-activated receptor alpha (PPARα), carnitine palmitoyltransferase 1a (CPT 1a), cytochrome P450-4A14 (CYP4A14), and cytochrome P450-4A10 (CYP4A10) in the livers of the four groups. N-V/N-Ali: normal (N) mice receiving vehicle (V) or aliskiren; MCD-V/MCD-Ali: MCD mice treated with vehicle or aliskiren #: p<0.05 vs. N-V; ##: p<0.01 vs. N-V; **: p<0.01 vs. MCD-V.

Aliskiren had no significant effects on the expression of lipogenic genes such as sterol regulatory element binding protein-1, carbohydrate responsive element binding protein, and peroxisome proliferator-activated receptor gamma. In contrast, aliskiren increased hepatic expression of fatty acid transport protein 1 and 4 (FATP1, FATP4) and stimulated fatty acid oxidation-related genes such as peroxisome proliferator-activated receptor alpha (PPAR-α), carnitine palmitoyltransferase 1a (CPT 1a), cytochrome P450-4A14 (CYP4A14). In the N-Ali group, the transcript expression of CYP4A10 or CYP4A14 was increased significantly compared with that in the N-V group (Figure 2). Furthermore, in the HepG2 cells treated with Ang II, the transcript expression of PPARα was decreased significantly (p<0.01). After adding aliskiren in the HepG2 cells treated with Ang II, the PPARα expression increased significantly (p=0.015) (Figure S1B in File S1). 

**Figure 3:**
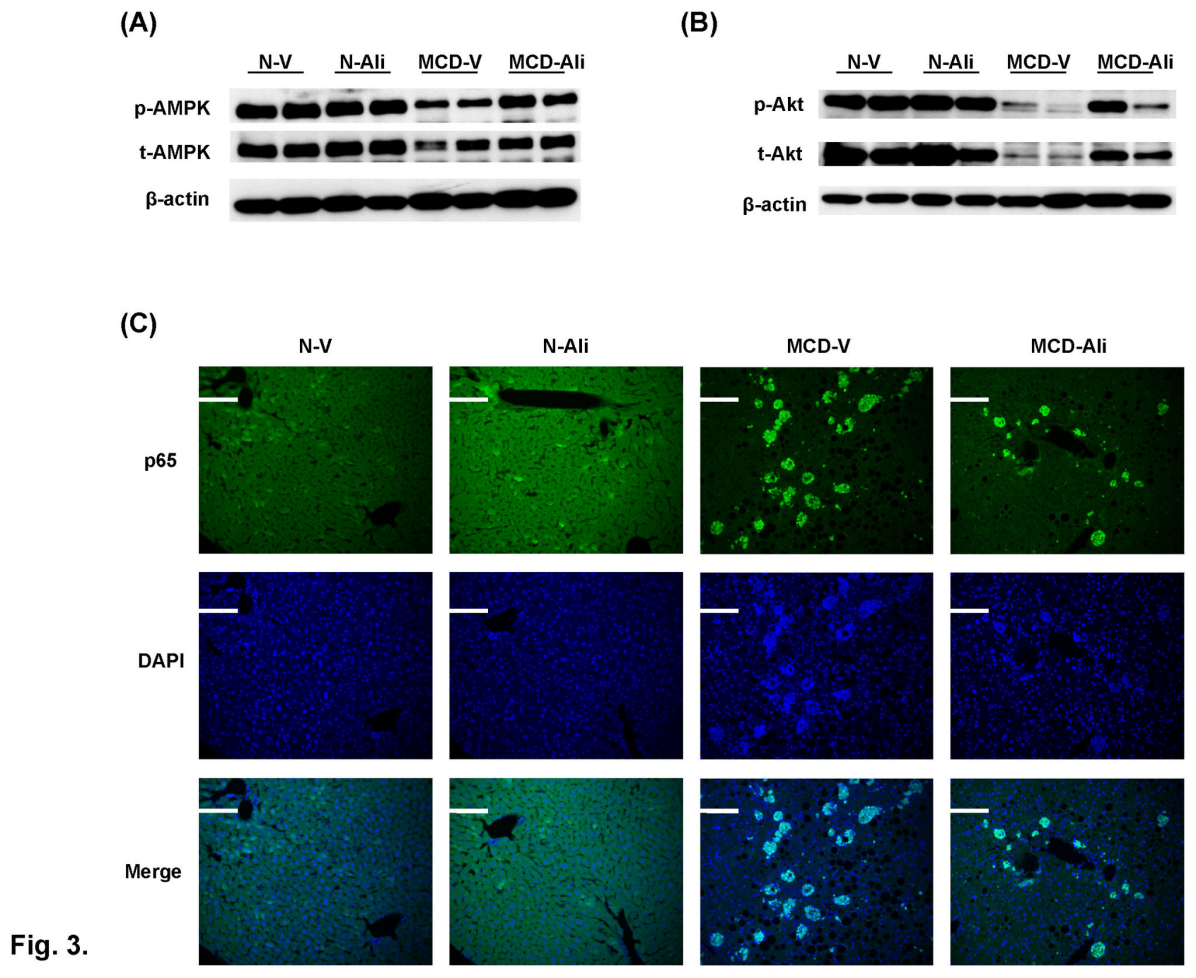
Aliskiren (Ali) up-regulated phosphorylation of AMPK and Akt but down-regulated expression of NF-κB p65 protein in mice fed with a methionine choline-deficient (MCD) diet. Western blotting of hepatic phosphorylated/total AMP-activated protein kinase (p-AMPK/t-AMPK) (**A**) and Akt (p-Akt/t-Akt) (**B**). (**C**) Representative images of nuclear translocation of p65 in the four groups. N-V/N-Ali: normal (N) mice receiving vehicle (V) or aliskiren; MCD-V/MCD-Ali: MCD mice treated with vehicle or aliskiren. Scale bar = 100 μm.

The phosphorylated AMP-activated protein kinase (AMPK) in the livers of the MCD-V group was slightly lower than that in the N-V group. Aliskiren significantly increased the expression of phosphorylated AMPK in the MCD-Ali mice (Figure 3A). Similarly, the phosphorylated Akt in livers of the MCD-V group was significantly lower than that in the N-V group. After aliskiren treatment, the phosphorylated Akt expression in the livers of the MCD-Ali mice was significantly increased (Figure 3B). 

### Aliskiren decreased hepatic expression of nuclear factor κB (NF-κB)

The activation of NF-κB p65 and p50 proteins was significantly higher in the MCD mice than that in the normal mice (Figure 3C and Figure S2 in File S1). Aliskiren significantly reduced the hepatic expressions of p65 and p50 in the MCD-Ali mice.

### Aliskiren decreased oxidative stress, hepatocytes apoptosis and activation of Kupffer cells in livers of the MCD mice

**Figure 4:**
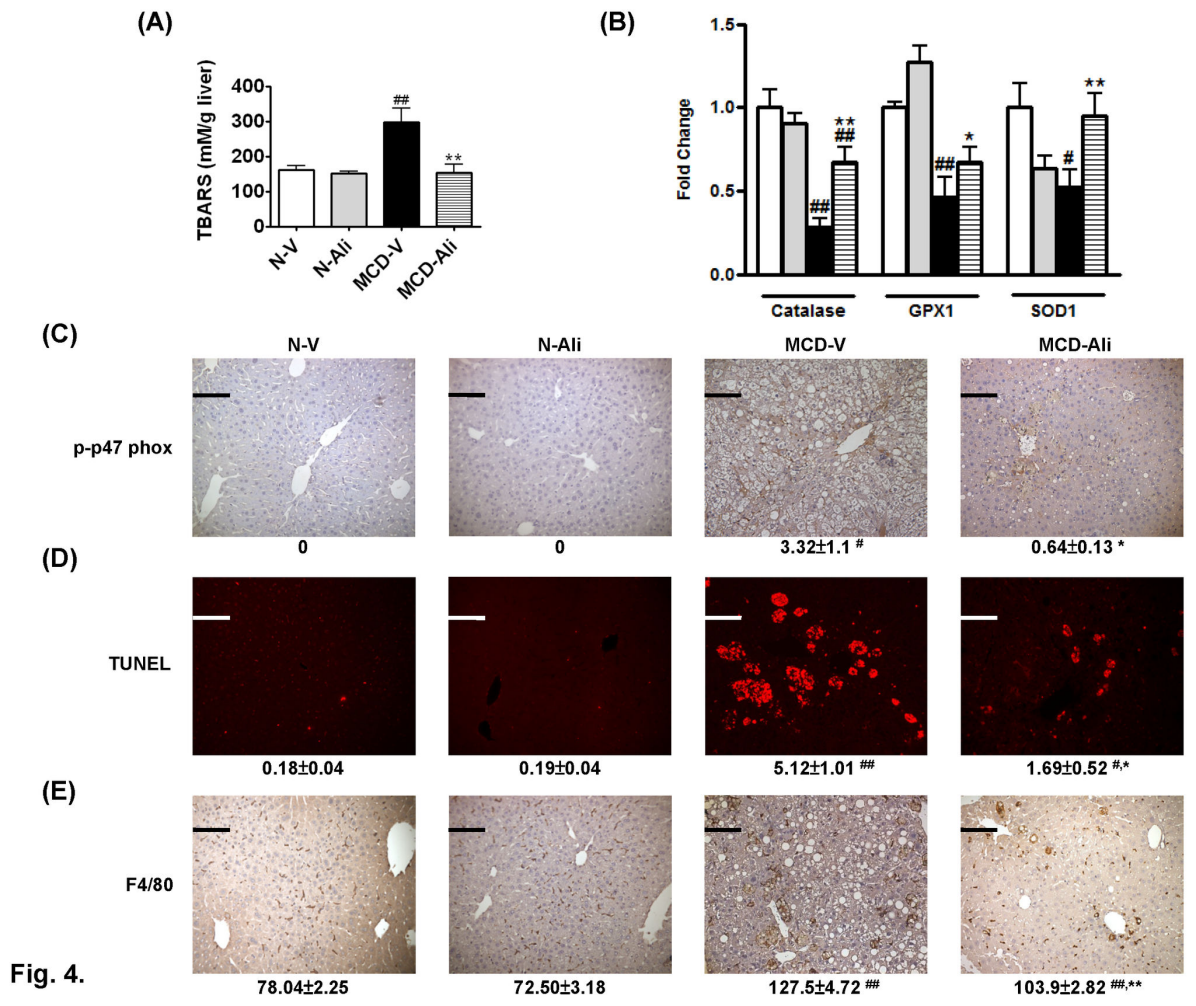
Aliskiren (Ali) reduced oxidative stress, hepatocyte apoptosis and Kupffer cells activation in mice fed with a methionine choline-deficient (MCD) diet. (**A**) The amount of thiobarbituric acid reactive substances (TBARS) and (**B**) expression of catalase, glutathione peroxidase-1 (GPX1) and superoxide dismutase-1 (SOD1) in the N-V (white bar), N-Ali (gray bar), MCD-V (black bar) and MCD-Ali (striped bar) groups. Representative images (×200) of phosphorylated p47 phox (p-p47 phox) (**C**), the terminal deoxynucleotide transferase mediated dUTP nick-end labeling (TUNEL) assay (**D**), and F4/80 (**E**) and quantification of positive stained areas (p-p47 phox and TUNEL) or cell counts (F4/80) per field of view to the below in all groups. Scale bar = 100 μm. N-V/N-Ali: normal (N) mice receiving vehicle (V) or aliskiren; MCD-V/MCD-Ali: MCD mice treated with vehicle or aliskiren. (#: P<0.05 vs. N-V; ##: P<0.01 vs. N-V; *: P<0.05 vs. MCD-V; **: P<0.01 vs. MCD-V).

The levels of TBARS (Figure 4A) and 4-Hydroxynonenal (4-HNE) (Figure S3A in File S1), the markers of increased oxidative stress, were significantly increased in the MCD-V mice. The mRNA expression of anti-oxidant enzymes including catalase, glutathione peroxidase-1 (GPX1) and superoxide dismutase-1 (SOD1) was significantly reduced in the livers of the MCD-V group (Figure 4B). The expression of p47 phox (Figure S3B in File S1) and phospho-p47 phox (Figure 4C) of nicotinamide adenine dinucleotide phosphate hydrogen oxidase was also increased significantly in the MCD-V group when compared with that in the N-V group. Aliskiren treatment not only significantly decreased the level of TBARS (Figure 4A), 4-HNE (Figure S3A in File S1), p47 phox (Figure S3B in File S1), phospho-p47 phox (Figure 4C) but also increased the expression of catalase 1, GPX1 and SOD1 (Figure 4B) in the mice fed with the MCD diet.

In livers of the MCD-V and MCD-Ali group, the stained apoptotic hepatocytes by TUNEL assay were significantly increased more than in the normal livers (Figure 4D). After aliskiren treatment, the apoptotic hepatocytes were significantly decreased in the MCD-Ali group. Furthermore, the amount of stained Kupffer cells (KCs) was reduced in the MCD-Ali mice (Figure 4E). Similarly, the expression of tumor necrosis factor alpha (TNF-α) was significantly increased in livers of the MCD-V group and attenuated in the MCD-Ali group (Figure S3C in File S1).

### The effects of aliskiren on liver fibrosis

**Figure 5:**
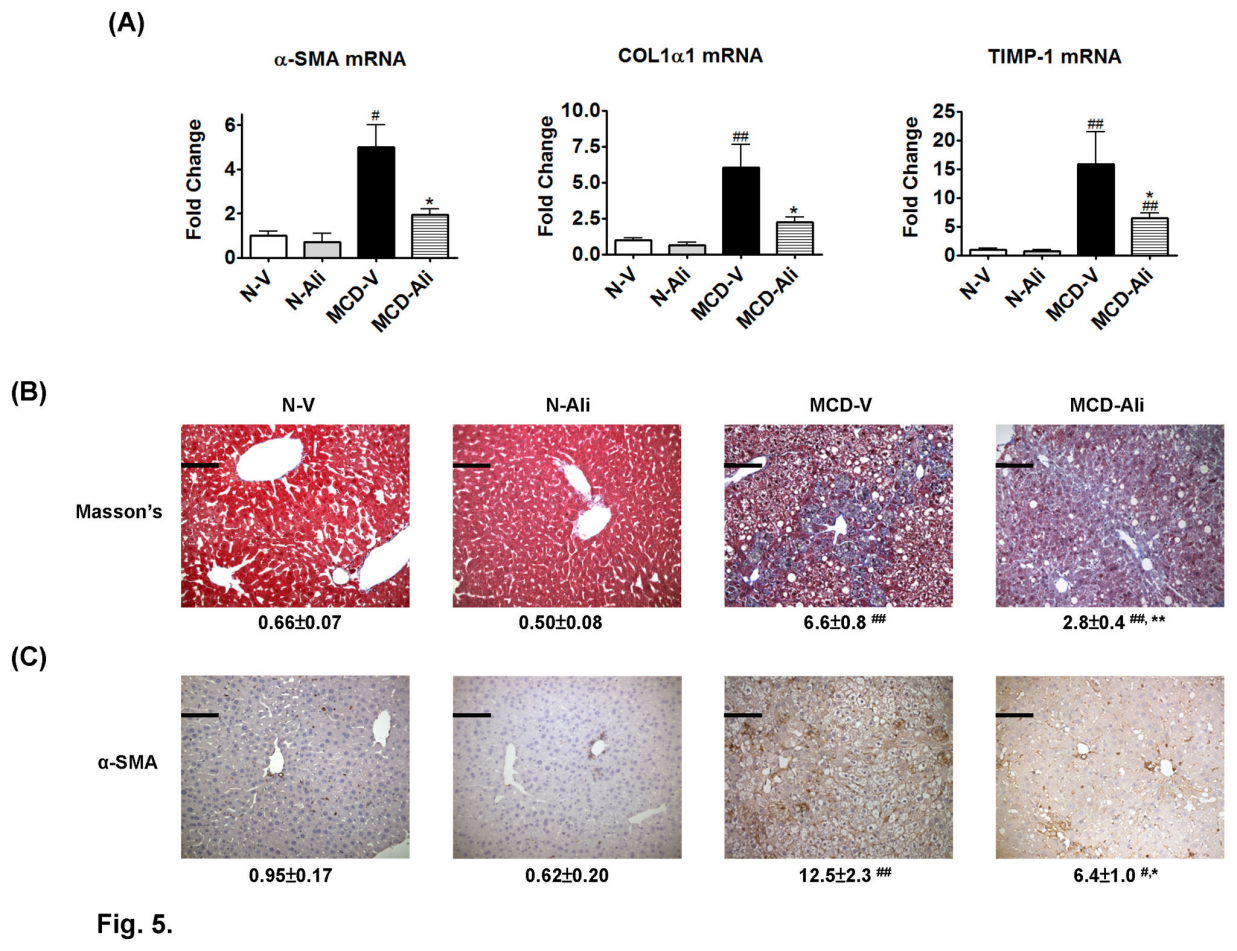
Aliskiren (Ali) reduced hepatic stellate cells activation, collagen deposition and gene expression of pro-fibrotic markers in mice fed with a methionine choline-deficient (MCD) diet. (**A**) Hepatic gene expression of alpha smooth muscle actin (α-SMA), collagen type I alpha 1 (COL1α1) and tissue inhibitor of metalloproteinases type I (TIMP-1) in the four groups. (**B**) Representative images (×200) of Masson's trichrome stain and α-SMA immunohistochemistry stain (**C**) in the livers. Scale bar = 100 μm. N-V/N-Ali: normal (N) mice receiving vehicle (V) or aliskiren; MCD-V/MCD-Ali: MCD mice treated with vehicle or aliskiren. (#: P<0.05 vs. N-V; ##: P<0.01 vs. N-V; *: P<0.05 vs. MCD-V; **: P<0.01 vs. MCD-V).

The hepatic expression of alpha smooth muscle actin (α-SMA), collagen type I alpha 1 (COL1α1) and tissue inhibitor of metalloproteinases type I (TIMP-1) was significantly higher in the MCD-V mice than that in the N-V mice (Figure 5A). In the MCD-Ali mice, the expression of α-SMA, COL1α1 and TIMP-1 was reduced significantly compared with that in the MCD-V mice.

The livers of the MCD-V and MCD-Ali mice exhibited more severe fibrosis and collagen deposition than did the normal livers. However, in the MCD-Ali mice, liver fibrosis, collagen deposition and amount of α-SMA positive cells were significantly reduced (Figures 1C, 5B, 5C; Table 2) when compared with those in the MCD-V group.

## Discussion

In previous research [7], renin knock-out mice were found to exhibit resistance to diet-induced obesity with the beneficial metabolic changes in these mice being reversed by angiotensin II administration. In our current study, the mice fed with MCD diet for 12 weeks manifested steatohepatitis and liver fibrosis with increased hepatic and systemic Ang II. Aliskiren treatment not only decreased inflammation and fibrosis but also steatosis with reduction of Ang II. Genetic deletion or pharmacologic inhibition of angiotensin converting enzyme [5] or angiotensin II type 1 receptor [3,4] has also been known to reduce hepatic steatosis in rodent models. However, the efﬁcacy of angiotensin-converting enzyme inhibitors and angiotensin receptor blockers in treating human NAFLD remains uncertain [16]; additionally, the effects of these drugs may be attenuated by the stimulation of renin resulting from the negative feedback loop because of the decreased Ang II activation [17]. Collectively, inhibition of renin activity by aliskiren is a potential way to clinically treat NAFLD, which urgently requires effective drugs [2]. 

The MCD mice receiving aliskiren manifested a decrease in hepatic lipid accumulation with increased expression of PPARα , CPT 1a, CYP4A10, CYP4A14, FATP1 and FATP4. This suggests that increased hepatic turnover of triglyceride occurred in these mice. PPARα has been known to increase hepatic uptake by up-regulating fatty acid transport proteins and breakdown of fatty acid by up-regulating CPT 1 and CYP4A [18]. In the HepG2 cells of our study, the steatosis was increased significantly by the incubation of Ang II with decreased expression of PPARα; the treatment with aliskiren attenuated steatosis with up-regulation of PPARα. Similarly, Nabeshima et al. [4] showed that deletion of angiotensin II type 1 receptor increased hepatic PPARα mRNA expression. These findings suggest that down-regulation of angiotensin II pathway might up-regulate PPARα. Moreover, in the livers of the N-Ali group, the expression of CYP4A10 and CYP4A14 was increased significantly without change in PPARα, CTP1a and Ang II. This indicates that aliskiren can independently induce the enzymes for omega oxidation of fatty acid. Therefore, aliskiren may increase hepatic turnover of triglyceride of the MCD mice by the synergistic effects of the up-regulated PPARα and CYP4A. 

Furthermore, the livers of the MCD-Ali group expressed more phosphorylation of AMPK. One possible cause of the activation of AMPK is up-regulated PPARα expression [19] in these mice. Another possibility is the up-regulation of FATP in the MCD-Ali mice which might also induce activation of AMPK [20]. AMPK activation not only inhibits hepatic glucose production but also leads to the stimulation of fatty acid oxidation and inhibition of lipogenesis [21]. Collectively, the increased activation of AMPK may have contributed to hypoglycemia and the reduced steatosis in the MCD-Ali group.

Rinella et al. have demonstrated that MCD mice exhibit a more pronounced response of blood glucose to insulin [22]. However, MCD mice have a decreased intrahepatic insulin resistance with reduced phosphorylation of Akt in response to an insulin stimulus [23]. Further, Ang II was observed to specifically inhibit activation of insulin-induced Akt in hepatocytes [24]. In our study, the MCD group showed decreased hepatic Akt phosphorylation and systemic insulin with increased Ang II. After aliskiren treatment, the levels of insulin and Ang II were decreased. Surprisingly, hepatic Akt phosphorylation increased significantly which implies that insulin induced Akt response was enhanced because of reduced inhibition of Ang II. 

Moreover, aliskiren increased systemic insulin sensitivity with hypoglycemia in the MCD mice. Our finding agrees with that observed by Marchionne et al. in obese Zucker rats [25] and Gandhi et al. in Streptozotocin-induced diabetic rats [26]. However, in other studies, aliskiren was found to improve insulin resistance but without reducing fasting blood glucose levels in db/db mice [11] and transgenic Ren2 rats [12,13]. Therefore, the improvement of insulin sensitivity after aliskiren treatment may be a general phenomenon. Nevertheless, aliskiren may have different modes of action in different animal models of glucose dysregulation. 

Recently, Kang et al. [11] administered aliskiren with a dose of 25mg/kg/day via mini pumps subcutaneously in db/db mice, which lacks the leptin receptor. They found no significant histologic improvement of hepatic steatosis in aliskiren-treated db/db mice. More notably, the hepatic triglyceride content of aliskiren-treated db/db mice was reduced about 50% to the level similar to that of the db/m mice. Aihara et al. [27] administered aliskiren orally with doses of 50 and 100 mg/kg/day in the choline-deficient L-amino acid-defined diet-induced rat, which is known to lack hepatic insulin resistance. They found that aliskiren did not significantly reduce hepatic steatosis. Lately, Kishina et al. [28] demonstrated decreased hepatic steatosis in fatty liver Shionogi-ob/ob male mice administered aliskiren orally with the dosage of 100 mg/kg/day; nevertheless, the underlying mechanism was not found out. Furthermore, in the HepG2 cells treated with aliskiren, we found that aliskiren decreased intracellular steatosis at a higher dose with up-regulation of PPARα, which was similar to the findings in our MCD-Ali group. In fact, aliskiren inhibits human, mouse, and rat plasma renin with an IC_50_ values of 0.6, 6, and 80 nmol/L respectively [14,29]. Therefore, a higher dose of aliskiren might be needed to significantly reduce hepatic steatosis. Indeed, the mechanism of steatosis is complex and different in each animal model. The anti-steatotic effect and involved mechanisms after aliskiren administration may not be identical in different animal models. Taken together, different species sensitivity to aliskiren, doses, models, and routes of administration may explain the discrepancy.

In this study, the oxidative stress was decreased after aliskiren treatment. Bataller et al. [30,31] have shown Ang II phosphorylated p47 phox and increased reactive oxygen species and end products of lipid peroxidation in the liver. The decrease in Ang II by aliskiren may have caused the down-regulation of hepatic p47 phox, and then the subsequent decrease of oxidative stress and production of end products of lipid peroxidation in the livers of the MCD-Ali group. Moreover, aliskiren administration could decrease lipoperoxide by up-regulating not only PPARα to deplete lipid but also CYP4A14 to degrade end products of lipid peroxidation [32]. Furthermore, the reverse of catalase, GPX1 and SOD1 after aliskiren treatment in the MCD mice may partly contribute to a decrease in oxidative stress. Aliskiren treatment induced up-regulation of PPARα, CYP4A14 and AMPK might be attributed to the up-regulation of these antioxidant enzymes in the livers [33,34]. 

In NAFLD, malondialdehyde and 4-HNE contribute to the progression of inflammation with increased production of proinflammatory cytokines, such as TNF-α and infiltration of inflammatory cells [35,36]. Additionally, these lipid peroxides not only activate hepatic stellate cells (HSCs) [37], causing collagen deposition and fibrosis, but also induce hepatocyte apoptosis [38,39]. The apoptotic bodies from hepatocytes engulfed by HSCs or KCs activate HSCs [40] and KCs [41], respectively. Therefore, the attenuation in liver inflammation, fibrosis, hepatocytes apoptosis, and activation of KCs and HSCs in the MCD-Ali group can be attributed to the reduction in oxidative stress by aliskiren treatment. 

NF-κB is known as a primary mediator of inflammation in experimental steatohepatitis [42]. In this study, aliskiren may have attenuated inflammation in part by inhibiting activation of NF-κB p65 and p50 in the MCD mice. The increased PPARα by aliskiren treatment may have contributed a role in inhibiting nuclear translocation of NF-κB [43]. Moreover, in the MCD mice, the increased Ang II could have activated NF-κB in hepatocytes [44]; and the increased oxidative stress and lipid peroxides could also have induced NF-κB activation [45,46]. Therefore, it is possible that aliskiren down-regulates NF-κB expression by up-regulating PPARα and decreasing levels of Ang II and oxidative stress in the liver. 

In conclusion, in addition to the beneficial effect of decreasing Ang II production, aliskiren can activate hepatic PPARα and AMPK to reduce hepatic steatosis and oxidative stress. The attenuation of the first hit and second hit by aliskiren treatment further improved liver inflammation and fibrosis in this MCD murine model. Aliskiren may thus be a potential therapeutic agent to treat human NASH. 

## Supporting Information

File S1 **Data supplement.** Includes Table S1 and S2 and Figure S1, S2, S3.
(DOC)
