# Supplementary material for: Aliskiren Attenuates Steatohepatitis and Increases Turnover of Hepatic Fat in Mice Fed with a Methionine and Choline Deficient Diet
Source: PLoS One. 2013 Oct 21;8(10):e77817. doi: 10.1371/journal.pone.0077817 (PMC3804600; doi:10.1371/journal.pone.0077817)
Supplement: File S1 — Data supplement. Includes Table S1 and S2 and Figure S1, S2, S3. (DOC) [file pone.0077817.s001.doc]

**DATA SUPPLEMENT**

**Aliskiren Attenuates Steatohepatitis and Increases Turnover of Hepatic Fat in Mice Fed with a Methionine and Choline Deficient Diet**

Kuei-Chuan Lee^1,2,3^, Che-Chang Chan^1,2,3^, Ying-Ying Yang^2,4^, Yun-Cheng Hsieh^1,2^, Yi-Hsiang Huang^1,2,3^, and Han-Chieh Lin^1,2^

^1^Division of Gastroenterology, Department of Medicine, Taipei Veterans General Hospital, Taipei, Taiwan; ^2^Department of Medicine, National Yang-Ming University School of Medicine, Taipei, Taiwan; ^3^Institute of Clinical Medicine, National Yang-Ming University School of Medicine, Taipei, Taiwan; ^4^Division of General Medicine, Department of Medicine, Taipei Veterans General Hospital, Taipei, Taiwan

**Corresponding Author:**

Han-Chieh Lin, M.D.

Division of Gastroenterology, Department of Medicine

Taipei Veterans General Hospital

#201, Section 2, Shih-Pai Road, Taipei 11217, Taiwan

Tel.: +886-2-2875-7506

Fax: +886-2-2873-9318

E-mail: hclin@vghtpe.gov.tw

Or

Yi-Hsiang Huang, M.D., Ph.D.

Institute of Clinical Medicine,

National Yang-Ming University School of Medicine, Taipei, Taiwan

#155, Section 2, Linong Street, Taipei 11221, Taiwan

Tel.: +886-2-2875-7403

Fax: +886-2-2874-5074

E-mail : yhhuang@vghtpe.gov.tw

**SUPPLMENTARY METHODS**

**Evaluating the anti-steatotic effect of aliskiren on HepG2 cells**

To measure whether aliskiren has a direct effect on steatosis of hepatocytes, we seeded HepG2 cells (obtained from Bioresource Collection and Research Center, Hsinchu, Taiwan) in 96-well culture dishes (10^4^ cells each well) for 24 hours. After removing the medium, the cells were washed twice with phosphate-buffered saline and incubated with HyClone Minimal Essential Medium with Earle's (α-MEM, Thermo Scientific) containing 10% bovine serum albumin for 48 hours. The medium was then added with vehicle, angiotensin II (Sigma, 10^-7^M) or aliskiren (10^-5^, 10^-7^ M) with or without angiotensin II (n=12 in each group) at 37°C for 24 hours. A dosage of 10^-5^M aliskiren had been found to completely inhibit renin activity in vitro . After incubation, the quantification of lipid accumulation each well was measured by a using steatosis colorimetric assay kit (Cayman) according to the manufacturer’s guidelines.

To obtain the RNA, HepG2 cells were seeded in 6-well culture dishes (3 × 10^5^ cells each well) for 24 hours. After removing the medium, the cells were washed twice with phosphate-buffered saline and incubated with α-MEM (Thermo Scientific) containing 10% bovine serum albumin for 24 hours. The vehicle, aliskiren (10^-5^M), angiotensin II (10^-7^M) or both of aliskiren (10^-5^M) and angiotensin II (10^-7^M) (n=6 in each group) was added into the medium during the incubation period. After incubation, the RNA of cells was extracted for real-time quantitative reverse transcriptase–polymerase chain reaction. The primers used are shown in supplementary Table 1. Relative expression target gene mRNA is normalized to the amount of β-actin mRNA.

**REFERENCES**

1. Ferri N, Greco CM, Maiocchi G, Corsini A (2011) Aliskiren reduces prorenin receptor expression and activity in cultured human aortic smooth muscle cells. J Renin Angiotensin Aldosterone Syst 12: 469-474.

**Supplementary Table 1. Primer pair**s used for quantitative real time PCR

| Gene name  For mouse | Primer | Sequence | Size |
| --- | --- | --- | --- |
| GPX1 | forward | 5'- TTACATTGTTTGAGAAGTGCGA -3' | 168 bp |
|  | reverse | 5'- CAAAGTTCCAGGCAATGTC -3' |  |
| Catalase | forward | 5'- TGAGAAGCCTAAGAACGCAATTC -3' | 63 bp |
|  | reverse | 5'- CCCTTCGCAGCCATGTG -3′ |  |
| SOD1 | forward | 5'- CATTCCATCATTGGCCGT -3' | 155 bp |
|  | reverse | 5'- TCAGACCACACAGGGAATGTTTA -3' |  |
| CYP4A10 | forward | 5′- GAGTGTCTCTGCTCTAAGCCCA-3′ | 443 bp |
|  | reverse | 5′- AGGCTGGGGTTAGCATCCTCCA-3′ |  |
| CYP4A14 | forward | 5′- CCCTGCTCCGCTTTGAATTG -3′ | 135 bp |
|  | reverse | 5′- AGCTGCCCTGACTCCATCA -3′ |  |
| SREBP1 | forward | 5′- AAGCAAATCACTGAAGGACCTGG -3′ | 150 bp |
|  | reverse | 5′- AAAGACAAGGGGCTACTCTGGGAG -3′ |  |
| ChREBP | forward | 5′- GGACAAGATCCGGCTGAACA -3′ | 131 bp |
|  | reverse | 5′- CAGGTTTCCGGTGCTCATCT -3′ |  |
| PPAR-ϒ | forward | 5′- TGGGTGAAACTCTGGGAGAT -3′ | 454 bp |
|  | reverse | 5′- CCATAGTGGAAGCCTGATGC -3′ |  |
| PPARα | forward | 5′- CCGAACATTGGTGTTCGCAG -3′ | 161 bp |
|  | reverse | 5′- AGATACGCCCAAATGCACCA -3′ |  |
| CPT1a | forward | 5′- CGCACGGAAGGAAAATGG -3′ | 211 bp |
|  | reverse | 5′- TGTGCCCAATATTCCTGG -3′ |  |
| FATP1 | forward | 5′- CGCTTTCTGCGTATCGTCTG -3′ | 120 bp |
|  | reverse | 5′- GATGCACGGGATCGTGTCT -3′ |  |
| FATP4 | forward | 5′- GATGGCCTCAGCTATCTGTGA -3′ | 202 bp |
|  | reverse | 5′- GGTGCCCGATGTGTAGATGTA -3′ |  |
| α-SMA | forward | 5′- GTCCCAGACATCAGGGAGTAA -3′ | 102 bp |
|  | reverse | 5′- TCGGATACTTCAGCGTCAGGA -3′ |  |
| TIMP-1 | forward | 5′- CCAGAGCCGTCACTTTGCTT -3′ | 126 bp |
|  | reverse | 5′- AGGAAAAGTAGACAGTGTTCAGGCTT -3′ |  |
| COL1α1 | forward | 5′- GCTTCTTTTCCTTGGGGTTC -3′ | 158 bp |
|  | reverse | 5′- GAGCGGAGAGTACTGGATCG -3′ |  |
| TNF-α | forward | 5′- CCAGTGTGGGAAGCTGTCTT -3′ | 100 bp |
|  | reverse | 5′- AAGCAAAAGAGGAGGCAACA -3′ |  |
| GAPDH | forward | 5′- TGTTGAAGTCGCAGGAGACAACCT -3′ | 111 bp |
|  | reverse | 5′- AACCTGCCAAGTATGATGACATCA -3′ |  |
| For HepG2 |  |  |  |
| PPARα | forward | 5′- GCGAACGATTCGACTCAAGC -3′ | 118 bp |
|  | reverse | 5′- CATCCCGACAGAAAGGCACT -3′ |  |
| CPT1a | forward | 5′- TTGCTGATGACGGCTATGGT -3′ | 100 bp |
|  | reverse | 5′- TGAGAATCCGTCTCAGGGCA -3′ |  |
| FATP4 | forward | 5′- GGCTCAGGGGCCAATAAACT -3′ | 148 bp |
|  | reverse | 5′- ACAGATGAGGCGGGTCAATG -3′ |  |
| β-actin | forward | 5′- AGAGCTACGAGCTGCCTGAC -3′ | 184 bp |
|  | reverse | 5′- AGCACTGTGTTGGCGTACAG -3′ |  |

**Supplementary Table 2. Antibody details and conditions used for Western blotting and immunohistochemistry**

| Antibody | Supplier | Catalog no. | Application | Dilution |
| --- | --- | --- | --- | --- |
| β-actin | Novus Biologicals | NB600-503 | WB | 1:5000 |
| Ang II | Phoenix pharmaceuticals | H-002-12 | IHC | 1:2000 |
| F4/80 | Abcam | Ab6640 | IHC | 1:400 |
| 4-HNE | Alpha Diagnostic | HNE11-S | IHC | 1:4000 |
| p47 phox | Novus biologicals | NBP1-61750 | IHC | 1:500 |
| p-p47 phox | Assaybiotech | P14598 | IHC | 1:400 |
| AMPK | Cell Signaling | #2532 | WB | 1:1000 |
| p-AMPK | Cell Signaling | #2535 | WB | 1:1000 |
| Akt | Cell Signaling | #9272 | WB | 1:1000 |
| p-Akt | Cell Signaling | #4058 | WB | 1:1000 |
| α-SMA | Millipore | #04-1094 | IHC | 1:200 |
| p50 | Santa cruz | sc-114x | IF | 1:2000 |
| p65 | Santa cruz | sc-7151x | IF | 1:2000 |

**Antibodies were purchased from Abcam (Cambridge, MA, U.S.A), Alpha Diagnostic International (San Antonio, Texas, U.S.A), Assay Biotechnology Co Inc (Sunnyvale, CA, U.S.A), Cell signaling (Beverley, MA, U.S.A), Millipore (MA, U.S.A), Novus biological (Littleton, CO, U.S.A), Phoenix pharmaceuticals (Burlingame, CA, U.S.A), Santa Cruz (CA, U.S.A). Ang II: Angiotensin II; 4-HNE: 4-Hydroxynonenal; p47 phox: neutrophil cytosol factor 1; AMPK: AMP-activated protein kinase; Akt: protein kinase B; α-SMA: alpha smooth muscle actin; WB: Western blotting; IHC: immunohistochemistry; IF: immunofluorescence.**

**Supplementary Figure 1**

**(A)**

**(B)**


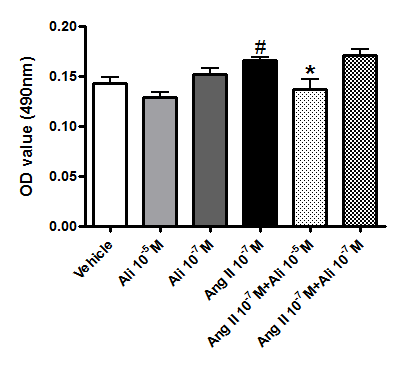

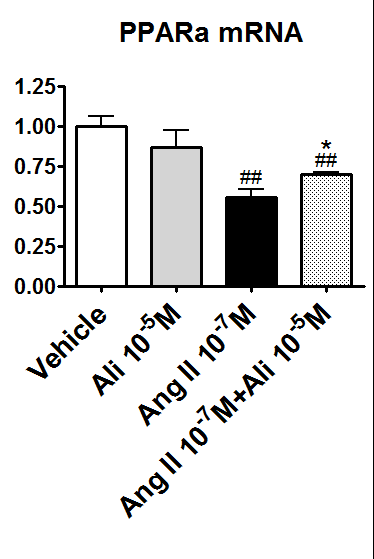

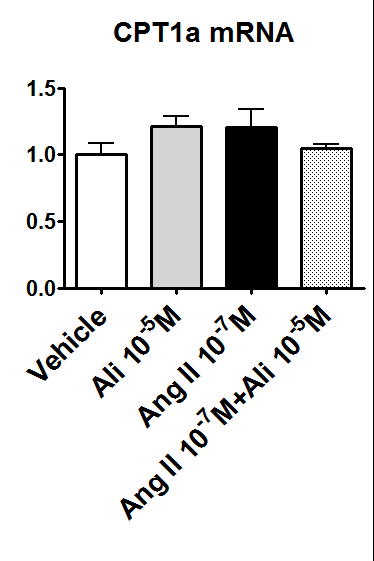

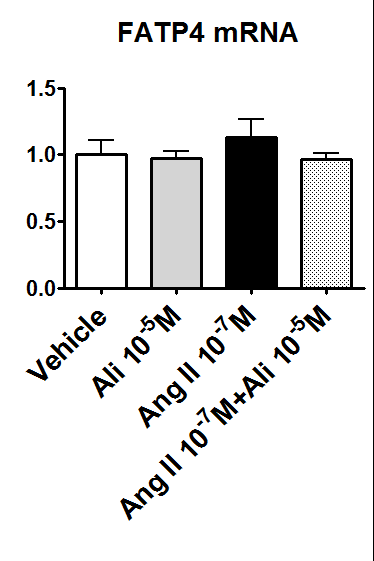


**Figure S1: Aliskiren inhibits steatosis in HepG2 cells.** **(A)** HepG2 cells (seeded 10^4^ cells per well) were treated with vehicle, angiotensin II (Ang II) or different doses of aliskiren (Ali) with or without Ang II (n=12 in each group). At the end of the experiment, the cells were stained with oil red O. Lipid accumulation was assessed by measuring the extracted oil red O at 450 nm. **(B)** The transcript expression of peroxisome proliferator-activated receptor alpha (PPARα), carnitine palmitoyltransferase 1a (CPT1a) and fatty acid transport protein 4 (FATP4) in HepG2 cells of the four groups (n=6 in each group). #: p<0.05 vs. the vehicle group; ##: p<0.01 vs. the vehicle group; *****: p<0.05 vs. the cells treated with Ang II 10^-7^M.

**Supplementary Figure 2**


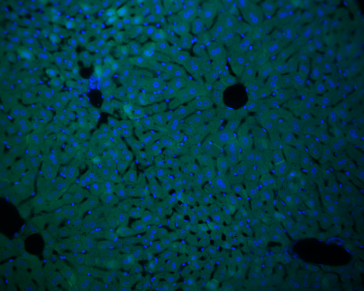

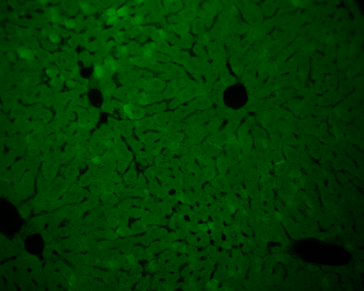

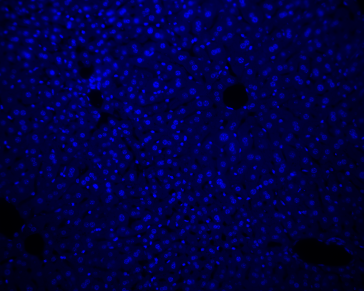

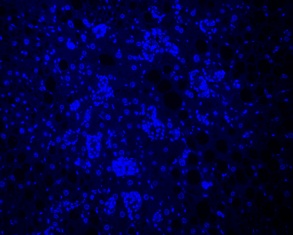

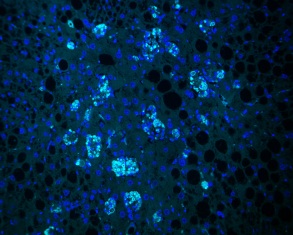

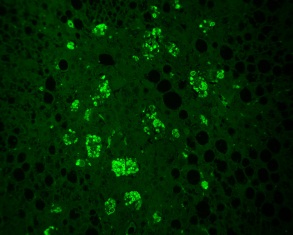

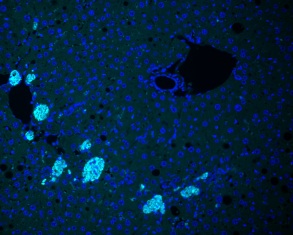

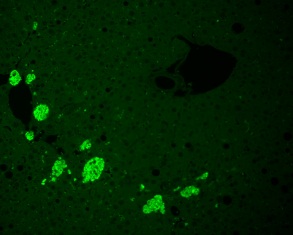

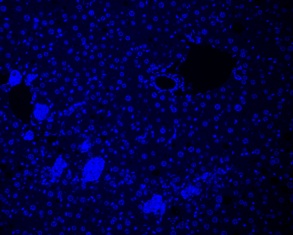


**p50**

**DAPI**

**Merge**

**N-V**

**MCD-V**

**MCD-Ali**


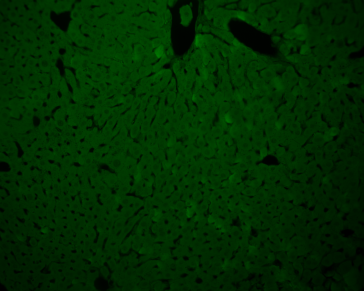

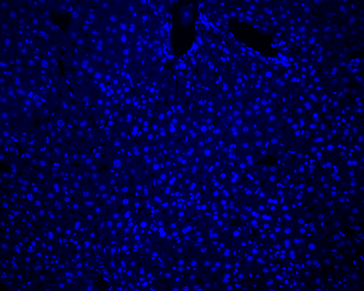

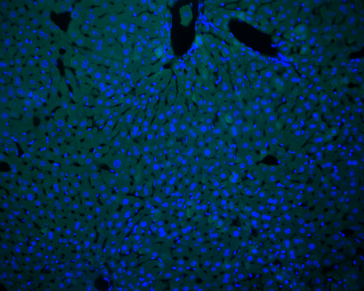


**N-Ali**

**Fig. S2. Aliskiren attenuated nuclear translocation of nuclear factor kappa B p50 protein in the MCD mice.** The representative images of immunofluorescence stain of p50 and 4',6-diamidino-2-phenylindole (DAPI) in the normal (N) and MCD mice receiving vehicle (V) or aliskiren (Ali). Scale bar: 100 μm.

**Supplementary Figure 3**

**(C)**


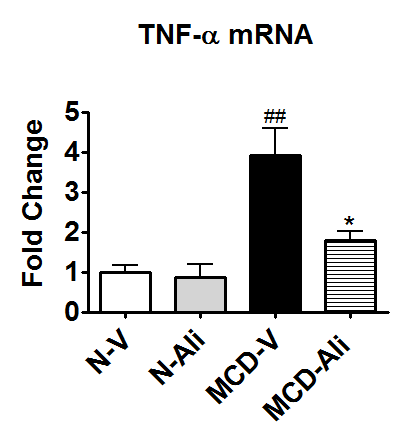


**(A)**

**(B)**


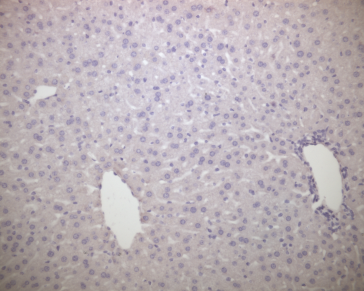

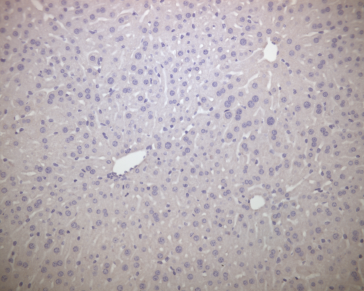

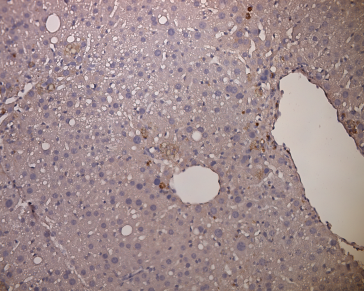

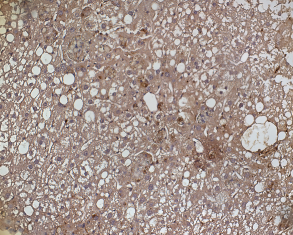


**4.16±0.7 ^##^**

**0.74±0.05 ****

**0**

**p47 phox**

**N-V**

**MCD-V**

**MCD-Ali**

**N-Ali**

**0**


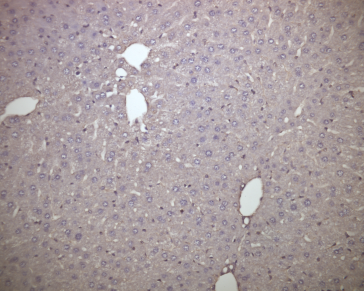

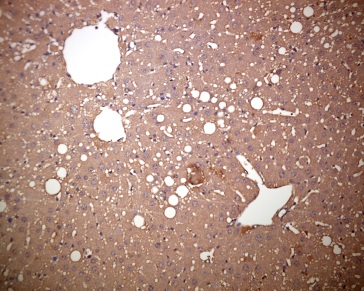

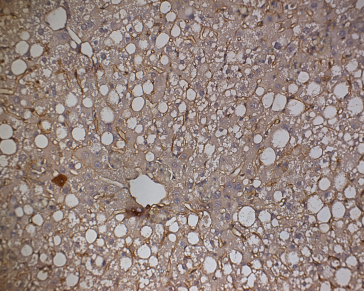


**N-V**

**MCD-V**

**MCD-Ali**

**4HNE**

**9.48±1.46 ^##^**

**1.24±0.77 ****

**0.42±0.12**

**N-Ali**


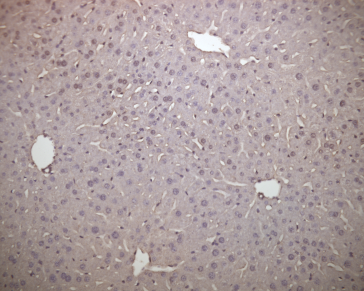


**0.44±0.13**

**Fig. S3. Aliskiren (Ali) decreased hepatic expression of 4-Hydroxynonenal (4-HNE), p47 phox of NADPH oxidase (p47 phox) and TNF-α in the MCD mice.** The immunohistochemistry images of 4-HNE **(A)** and p47 phox **(B)** in the four groups and quantification of positive stained areas per field of view to the below. **(C)** The transcript expression of tumor necrosis factor alpha (TNF-α) in all groups. N-V/N-Ali: normal (N) mice receiving vehicle (V) or aliskiren; MCD-V/MCD-Ali: MCD mice treated with vehicle or aliskiren. ##: p<0.01 vs. N-V; *****: p<0.05 vs. MCD-V; ******: p<0.01 vs. MCD-V.
